# Bidirectional association between gallstones and renal stones: Two longitudinal follow-up studies using a national sample cohort

**DOI:** 10.1038/s41598-019-38964-2

**Published:** 2019-02-22

**Authors:** So Young Kim, Chang Myeon Song, Hyun Lim, Man Sup Lim, Woojin Bang, Hyo Geun Choi

**Affiliations:** 1 Department of Otorhinolaryngology-Head & Neck Surgery, CHA Bundang Medical Center, CHA University, Seongnam, Korea; 2 Department of Otorhinolaryngology-Head & Neck Surgery, Hanyang University College of Medicine, Seoul, Korea; 3 Department of Internal Medicine, Hallym University College of Medicine, Anyang, Korea; 4 Department of General Surgery, Hallym University College of Medicine, Chuncheon, Korea; 5 Department of Urology, Hallym University College of Medicine, Anyang, Korea; 6 Department of Otorhinolaryngology-Head & Neck Surgery, Hallym University College of Medicine, Anyang, Korea

## Abstract

The present study evaluated the associations between gallstones and renal stones using a national sample cohort of the Korean population. The Korean National Health Insurance Service-National Sample Cohort was collected from 2002 to 2013. We designed two different longitudinal follow-up studies. In study I, we extracted gallstone patients (n = 20,711) and 1:4-matched control I subjects (n = 82,844) and analyzed the occurrence of renal stones. In study II, we extracted renal stone patients (n = 23,615) and 1:4-matched control II subjects (n = 94,460) and analyzed the occurrence of gallstones. Matching was performed for age, sex, income, region of residence, and history of hypertension, diabetes mellitus, and dyslipidemia. Crude and adjusted hazard ratios (HRs) were calculated using a Cox proportional hazards model, and the 95% confidence intervals (CIs) were calculated. Subgroup analyses were performed according to age and sex. The adjusted HR of renal stones was 1.93 (95% CI = 1.75–2.14) in the gallstone group (P < 0.001). The adjusted HR of gallstones was 1.97 (95% CI = 1.81–2.15) in the renal stone group (P < 0.001). The results were consistent in all subgroup analyses. Gallstones increased the risk of renal stones, and renal stones increased the risk of gallstones.

## Introduction

A gallstone is a crystalline deposit in the gallbladder^1^. The prevalence of gallstones is 5.5% in men and 8.6% in women in the USA^2^ and 4.2–5.3% in Korea^3,4^. Gallstones are categorized as cholesterol stones, pigment stones, or mixed stones based on their composition^1^. Their prevalence rates in Korea are 58.1% for cholesterol stones, 25.2% for black pigment stones, and 12.1% for brown pigment stones^5^. Age, female sex, ethnicity, estrogen treatment, obesity, Western diet, low physical activity, liver cirrhosis, diabetes mellitus, and dyslipidemia are known risk factors for gallstones^6^.

A renal stone is a stone in the kidney or lower urinary tract. The prevalence of renal stones is 10.6% in men and 7.1% women in the USA^7^ and 5.0% in Korea^8^. The annual incidence is estimated to be 457 per 100,000 people in Korea^9^. The exact pathophysiology of renal stone formation is unclear. Various risk factors have been proposed and include chronic kidney disease, poor hydration, abnormalities in calcium metabolism including hyperparathyroidism, increasing age, obesity, diabetes mellitus, warm climate, and high animal protein intake^10–13^. Approximately 80% of kidney stones are composed of calcium substrates. Renal stones are composed of calcium oxalate (56–61%), calcium phosphate (8–18%), uric acid (9–17%), struvite, or cysteine^13^.

The association between gallstones and renal stones has been reported in several studies^14–16^. However, the direction of the effect cannot be sufficiently evaluated in a cross-sectional study^16^. Only a single direction of the effect was evaluated in a cohort study^14^. Several studies have evaluated the effect using a bidirectional approach^15^. The purpose of this study is to bidirectionally evaluate the association between gallstones and renal stones using a national sample cohort of the Korean population. We designed two different longitudinal follow-up studies. In one study, we extracted gallstone patients and 1:4-matched control subjects and analyzed the occurrence of renal stones. In the other study, we extracted renal stone patients and 1:4-matched control subjects and analyzed the occurrence of gallstones.

## Results

### Study I

The mean follow-up periods were 65.74 (standard deviation, SD = 41.60) months and 64.87 (SD = 41.80) months for the gallstone group and the control I group, respectively. The rate of renal stones was higher in the gallstone group (2.7% [563/20,711]) than in the control I group (1.4% [1,180/82,844], P < 0.001, Table 1). The general characteristics (age, sex, income, region of residence, and hypertension, diabetes, and dyslipidemia histories) of participants were exactly the same due to the matching (P = 1.000).

**Table 1:** General Characteristics of Participants.

Characteristics	Study I			Study II		
	Gallstone (n, %)	Control I (n, %)	P-value	Renal stone (n, %)	Control II (n, %)	P-value
Age (years old)			1.000			1.000
0–4	15 (0.1)	60 (0.1)		15 (0.1)	60 (0.1)	
5–9	9 (0.0)	36 (0.0)		29 (0.1)	116 (0.1)	
10–14	29 (0.1)	116 (0.1)		91 (0.4)	364 (0.4)	
15–19	171 (0.8)	684 (0.8)		443 (1.9)	1,772 (1.9)	
20–24	292 (1.4)	1,168 (1.4)		845 (3.6)	3,380 (3.6)	
25–29	684 (3.3)	2,736 (3.3)		1,595 (6.8)	6,380 (6.8)	
30–34	1,164 (5.6)	4,656 (5.6)		2,307 (9.8)	9,228 (9.8)	
35–39	1,505 (7.3)	6,020 (7.3)		2,740 (11.6)	10,960 (11.6)	
40–44	1,867 (9.0)	7,468 (9.0)		2,947 (12.5)	11,788 (12.5)	
45–49	2,073 (10.0)	8,292 (10.0)		3,068 (13.0)	12,272 (13.0)	
50–54	2,341 (11.3)	9,364 (11.3)		2,809 (11.9)	11,236 (11.9)	
55–59	2,160 (10.4)	8,640 (10.4)		2,279 (9.7)	9,116 (9.7)	
60–64	2,197 (10.6)	8,788 (10.6)		1,826 (7.7)	7,304 (7.7)	
65–69	1,988 (9.6)	7,952 (9.6)		1,304 (5.5)	5,216 (5.5)	
70–74	1,798 (8.7)	7,192 (8.7)		766 (3.2)	3,064 (3.2)	
75–79	1,262 (6.1)	5,048 (6.1)		360 (1.5)	1,440 (1.5)	
80–84	755 (3.6)	3,020 (3.6)		145 (0.6)	580 (0.6)	
85+	401 (1.9)	1,604 (1.9)		46 (0.2)	184 (0.2)	
Sex			1.000			1.000
Male	10,027 (48.4)	40,108 (48.4)		15,260 (64.6)	61,040 (64.6)	
Female	10,684 (51.6)	42,736 (51.6)		8,355 (35.4)	33,420 (35.4)	
Income			1.000			1.000
1 (lowest)	634 (3.1)	2,536 (3.1)		284 (1.2)	1,136 (1.2)	
2	1,468 (7.1)	5,872 (7.1)		1,386 (5.9)	5,544 (5.9)	
3	1,200 (5.8)	4,800 (5.8)		1,484 (6.3)	5,936 (6.3)	
4	1,254 (6.1)	5,016 (6.1)		1,646 (7.0)	6,584 (7.0)	
5	1,466 (7.1)	5,864 (7.1)		1,697 (7.2)	6,788 (7.2)	
6	1,623 (7.8)	6,492 (7.8)		1,993 (8.4)	7,972 (8.4)	
7	1,835 (8.9)	7,340 (8.9)		2,384 (10.1)	9,536 (10.1)	
8	2,147 (10.4)	8,588 (10.4)		2,649 (11.2)	10,596 (11.2)	
9	2,486 (12.0)	9,944 (12.0)		2,992 (12.7)	11,968 (12.7)	
10	2,956 (14.3)	11,824 (14.3)		3,341 (14.1)	13,364 (14.1)	
11 (highest)	3,642 (17.6)	14,568 (17.6)		3,759 (15.9)	15,036 (15.9)	
Region of residence			1.000			1.000
Urban	9,290 (44.9)	37,160 (44.9)		11,149 (47.2)	44,596 (47.2)	
Rural	11,421 (55.1)	45,684 (55.1)		12,466 (52.8)	49,864 (52.8)	
Hypertension			1.000			1.000
Yes	9,008 (43.5)	36,032 (43.5)		8,367 (35.4)	33,468 (35.4)	
No	11,703 (56.5)	46,812 (56.5)		15,248 (64.6)	60,992 (64.6)	
Diabetes Mellitus			1.000			1.000
Yes	5,366 (25.9)	21,464 (25.9)		4,509 (19.1)	18,036 (19.1)	
No	15,345 (74.1)	61,380 (74.1)		19,106 (80.9)	76,424 (80.9)	
Dyslipidemia			1.000			1.000
Yes	6,620 (32.0)	26,480 (32.0)		6,850 (29.0)	27,400 (29.0)	
No	14,091 (68.0)	56,364 (68.0)		16,765 (71.0)	67,060 (71.0)	
Renal stone			<0.001*			
Yes	563 (2.7)	1,180 (1.4)		N/A	N/A	
No	20,148 (97.3)	81,664 (98.6)		N/A	N/A	
Gallstone						<0.001*
Yes	N/A	N/A		793 (3.4)	1,631 (1.7)	
No	N/A	N/A		22,822 (96.6)	92,829 (98.3)	

^*^Chi-square test, significance at P < 0.05.

The crude and adjusted HRs of renal stones were 1.93 (95% CI = 1.75–2.14) and 1.93 (95% CI = 1.75–2.14) in the gallstone group, respectively (each P < 0.001, Table 2). In subgroup analyses, all crude and adjusted HRs of renal stones were higher in the gallstone group (each P < 0.05, Table 3). The adjusted HRs were 2.23 (95% CI = 1.10–4.50) in men <30 years old; 2.68 (1.20–5.96) in women <30 years old; 1.90 (95% CI = 1.62–2.23) in men 30–59 years old; 2.31 (95% CI = 1.89–2.82) in women 30–59 years old; 1.62 (95% CI = 1.27–2.06) in men ≥60 years old; and 1.78 (95% CI = 1.36–2.33) in women ≥60 years old.

**Table 2:** Crude and adjusted hazard ratios (95% confidence intervals) of gallstone for renal stone in study I.

Characteristics	Renal stone			
	Crude	P-value	Adjusted^†^	P-value
Gallstone	1.93 (1.75–2.14)	<0.001^*^	1.93 (1.75–2.14)	<0.001^*^
Control	1.00		1.00	

^*^Cox proportional hazard regression model, significance at P < 0.05.

^†^Adjusted model for age, sex, income, region of residence, hypertension, diabetes, and dyslipidemia.

**Table 3:** Subgroup analysis of crude and adjusted hazard ratios (95% confidence intervals) of gallstone for renal stone according to age and sex in study I.

Characteristics	Renal stone			
	Crude	P-value	Adjusted^†^	P-value
Age <30 years old, men (n = 2,395)				
Gallstone	2.22 (1.10–4.48)	0.027^*^	2.23 (1.10–4.50)	0.026^*^
Control	1.00		1.00	
Age <30 years old, women (n = 3,605)				
Gallstone	2.68 (1.20–5.96)	0.016*	2.68 (1.20–5.96)	0.016*
Control	1.00		1.00	
Age ≥30 and <60 years old, men (n = 27,720)				
Gallstone	1.90 (1.62–2.22)	<0.001*	1.90 (1.62–2.23)	<0.001*
Control	1.00		1.00	
Age ≥30 and <60 years old, women (n = 27,830)				
Gallstone	2.30 (1.89–2.81)	<0.001*	2.31 (1.89–2.82)	<0.001*
Control	1.00		1.00	
Age ≥60 years old, men (n = 20,020)				
Gallstone	1.62 (1.27–2.06)	<0.001*	1.62 (1.27–2.06)	<0.001*
Control	1.00		1.00	
Age ≥60 years old, women (n = 21,985)				
Gallstone	1.78 (1.36–2.34)	<0.001*	1.78 (1.36–2.33)	<0.001*
Control	1.00		1.00	

*Cox proportional hazard regression model, significance at P < 0.05.

^†^Adjusted model for age, sex, income, region of residence, hypertension, diabetes, and dyslipidemia.

### Study II

The mean follow-up periods were 72.25 (SD = 41.52) months and 71.31 (SD = 41.75) months for the renal stone group and the control II group, respectively. The rate of gallstones was higher in the renal stone group (3.4% [793/23,615]) than in the control II group (1.7% [1,631/94,460], P < 0.001, Table 1). The general characteristics (age, sex, income, region of residence, and hypertension, diabetes, and dyslipidemia histories) of participants were exactly the same due to the matching (P = 1.000).

The crude and adjusted HRs of gallstones were 1.97 (95% CI = 1.81–2.14) and 1.97 (95% CI = 1.81–2.15) in the renal stone group, respectively (each P < 0.001, Table 4). In the subgroup analyses, all crude and adjusted HRs of gallstones were higher in the renal stone group (each P < 0.05, Table 5). The adjusted HRs were 1.78 (95% CI = 1.05–3.02) in men <30 years old; 3.27 (95% CI = 1.72–6.20) in women <30 years old; 1.85 (95% CI = 1.62–2.11) in men 30–59 years old; 2.43 (95% CI = 2.03–2.91) in women 30–59 years old; 1.63 (95% CI = 1.32–2.00) in men ≥60 years old; and 2.15 (95% CI = 1.71–2.69) in women ≥60 years old.

**Table 4:** Crude and adjusted hazard ratios (95% confidence intervals) of renal stone for gallstone in study II.

Characteristics	Gallstone			
	Crude	P-value	Adjusted^†^	P-value
Renal stone	1.97 (1.81–2.14)	<0.001*	1.97 (1.81–2.15)	<0.001*
Control	1.00		1.00	

*Cox proportional hazard regression model, significance at P < 0.05.

^†^Adjusted model for age, sex, income, region of residence, hypertension, diabetes, and dyslipidemia.

**Table 5:** Subgroup analysis of crude and adjusted hazard ratios (95% confidence intervals) of renal stone for gallstone according to age and sex in study II.

Characteristics	Gallstone			
	Crude	P-value	Adjusted^†^	P-value
Age <30 years old, men (n = 10,480)				
Renal stone	1.78 (1.05–3.02)	0.032*	1.78 (1.05–3.02)	0.032*
Control	1.00		1.00	
Age <30 years old, women (n = 4,610)				
Renal stone	3.26 (1.72–6.18)	<0.001*	3.27 (1.72–6.20)	<0.001*
Control	1.00		1.00	
Age ≥30 and <60 years old, men (n = 53,690)				
Renal stone	1.85 (1.62–2.11)	<0.001*	1.85 (1.62–2.11)	<0.001*
Control	1.00		1.00	
Age ≥30 and <60 years old, women (n = 27,060)				
Renal stone	2.43 (2.03–2.91)	<0.001*	2.43 (2.03–2.91)	<0.001*
Control	1.00		1.00	
Age ≥60 years old, men (n = 12,130)				
Renal stone	1.62 (1.32–1.99)	<0.001*	1.63 (1.32–2.00)	<0.001*
Control	1.00		1.00	
Age ≥60 years old, women (n = 10,105)				
Renal stone	2.15 (1.72–2.69)	<0.001*	2.15 (1.71–2.69)	<0.001*
Control	1.00		1.00	

^*^Cox proportional hazard regression model, significance at P < 0.05.

^†^Adjusted model for age, sex, income, region of residence, hypertension, diabetes, and dyslipidemia.

## Discussion

This study revealed a bidirectional association between gallstones and renal stones. Gallstones increased the risk of renal stones, and renal stones increased the risk of gallstones. In both studies, these relationships were consistent in all subgroups according to age and sex.

The results of this study are similar to those of previous studies. Previous cohort studies have reported an increased HR of renal stones in gallstone patients as follows: 1.68 (95% = 1.59–1.77) in the general population^14^ and 1.26 (95% CI = 1.09–1.44) in older women, 1.32 (95% CI = 1.14–1.52) in young women, and 1.28 (95% CI 1.03–1.57) in men^15^. The HR was 1.93 (95% CI = 1.75–2.14) in study I. The HR of gallstones in renal stone patients has been reported as follows: 1.17 (95% CI = 1.06–1.29) in older women, 1.31 (95% CI = 1.19–1.45) in young women, and 1.51 (95% CI = 1.35–1.68) in men^15^. The HR was 1.95 (95% CI = 1.18–2.15) in study II. In a cross-sectional design study, the odds ratio (OR) between renal stones and gallstones has been reported as follows: 1.65 (95% CI = 1.46–1.86) in older women, 1.85 (95% CI = 1.65–2.07) in young women, 1.61 (95% CI = 1.41–1.85) in men^15^ and 1.46 (95% CI = 1.24–1.73) in white individuals, and 3.01 (1.86–4.88) in African Americans^16^.

The association between gallstones and renal stones could be explained by common pathophysiology. First, obesity and metabolic syndrome are risk factors for both gallstones and renal stones^17,18^. Hyperinsulinemia may increase hepatic cholesterol secretion and cholesterol supersaturation by activating hydroxymethylglutaryl coenzyme A reductase or by upregulating hepatocyte LDL receptors^19,20^. Obesity and insulin resistance result in defective ammoniagenesis^21^, so diabetes can increase the risk of uric acid renal stones due to a low urinary pH^22^. Second, obesity is an independent risk factor for infection^23^. Infection of the gallbladder can result in liver cell damage and bile acid excretion^24^. Hemolysis and chronic bacterial infections can produce pigment stones in the gallbladder^25^. Obesity can increase the risk of urinary tract infections^26^. Third, intestinal malabsorption can result in decreased bile acid resorption and increased urinary oxalate^27^. Finally, water and ion channels in the gallbladder might affect urine composition^28^.

The advantages of this study are consistent with those of our previous studies using the national sample cohort^29–31^. We used a large, representative, nationwide population. Because National Health Insurance Service (NHIS) data include all citizens in the nation without exception, no participants were missing during the follow-up periods. The control groups were randomly selected by matching for age, sex, income, region of residence, and past medical histories to avoid confounding effects. Furthermore, an adjusted hazard model was used to minimize the confounders. We designed two different studies to analyze the direction of the effect.

This study has the several limitations. Despite the cohort study design, we could not exclude the effects of possible confounders that might affect both gallstones and renal stones. Because we did not have data for body mass index or smoking or alcohol history, we could not adjust for these factors. These lifestyle factors could influence or mediate the association between gallstones and renal stones. Patients who could not consult with a clinic might have been missed. The possibility of detection bias exists. Visits for one disease could increase the detection rates of the other disease. Therefore, we performed an additional analysis confined to >6 months after the detection of one disease (Supplementary Table S1). In study I, we analyzed the occurrence of renal stones >6 months after detection of a gallstone. The adjusted HR was 1.43 (95% CI = 1.27–1.61, P < 0.001). In study II, we analyzed the occurrence of gallstones >6 months after detection of a renal stone. The adjusted HR was 1.53 (95% CI = 1.39–1.69, P < 0.001). Therefore, the association between gallstones and renal stones was consistent, even considering the possibility of detection bias.

In conclusion, gallstones increased the risk of renal stones, and renal stones increased the risk of gallstones.

## Materials and Methods

### Study Population and Data Collection

This study was approved by the ethics committee of Hallym University (2014-I148). The ethics committee of Hallym University waived the written informed consent from the study participants. All analyses adhered to the guidelines and regulations of the ethics committee of Hallym University.

This national cohort study used data of the Korean Health Insurance Review and Assessment Service-National Sample Cohort (HIRA-NSC). The sample cohort was directly extracted from the mother population from the Korean NHIS to minimize non-sampling errors. The sample cohort consisted of approximately 2% of the entire Korean population (50 million). The sampling was performed based on the 1,476 levels (age [18 categories], sex [2 categories], and income level [41 categories]) using randomized stratified systematic sampling methods via proportional allocation to represent the entire population. After data selection, a statistician verified the appropriateness of the sample by comparing the data from the entire Korean population to the sample data. The National Health Insurance Sharing Service provided the details of the sampling procedures on their website^32^. This cohort database is composed of (i) personal information, (ii) health insurance claim codes (procedures and prescriptions), (iii) diagnostic codes using the International Classification of Disease-10 (ICD-10), (iv) death records from the Korean National Statistical Office (using the Korean Standard Classification of disease), (v) socio-economic data (residence and income), and (vi) medical examination data for each participant over 2002 to 2013.

The exact population statistics were available using the NHIS database because all Korean citizens are identified by a 13-digit resident registration number from birth to death. All Koreans have to enroll in the NHIS. All the medical records of all Korean hospitals and clinics are registered using the 13-digit resident registration number to register individual patients in the medical insurance system. Thus, the risk of duplicated medical records is minimal, even if a patient visits different hospitals or clinics. In addition, all medical treatments in Korea can be traced without exclusion using the HIRA system. In Korea, reporting notice of death to an administrative entity is legally obligatory before a funeral can be held, and the reason of death and date are documented by medical doctors on a death certificate.

### Participant Selection

Of 1,125,691 patients with 114,369,638 medical claim codes, the participants who were diagnosed with gallstones (ICD-10: K80; Cholelithiasis) were included. Among them, the participants who visited hospitals or clinics ≥2 times for gallstones (n = 21,501) were selected. Histories of renal stones were classified using ICD-10 codes (N20; Calculus of kidney and ureter). We selected participants who visited hospitals or clinics ≥2 times for renal stones (n = 24,123).

**Figure 1:**
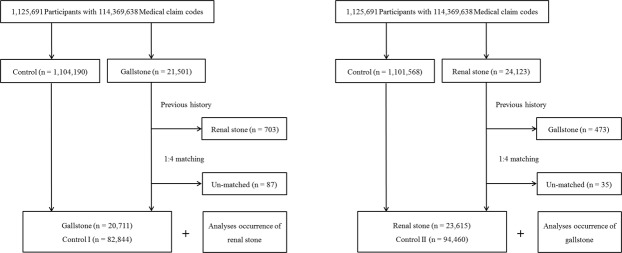
Schematic illustration of the participant selection process that was used in the present study. (**a**) Of a total of 1,125,691 participants, 20,711 gallstone patients were matched with 82,844 control I participants for age, group, sex, income group, region of residence, and past medical histories. (**b**) Of a total 1,125,691 participants, 23,165 renal stone patients were matched with 94,460 control II participants for age, group, sex, income group, region of residence, and past medical histories.

We designed study I and II. These studies are independent from each other. For study I, the patients with gallstone were followed up for the presence of renal stone (Fig. 1a). Thus, gallstone was the first stone event, followed by renal stone. In contrast, study II investigated the subsequent occurrence of gallstone after renal stone (Fig. 1b). In these cases, the renal stone was the first stone event, followed by gallstone.

#### Study I

The 1:4 matching was performed between the gallstone patients and the control I group who were never diagnosed with gallstones from 2002 through 2013. The control group was selected from the total population (n = 1,104,190). The age, group, sex, income group, region of residence, and past medical histories (hypertension, diabetes, and dyslipidemia) were matched between the gallstone and control groups. The selection bias was minimized by sorting the control I participants using a random number order and then selecting them from top to bottom. It was presumed that the matched control I participants were involved at the same time as each matched gallstone participant (index date). Thus, participants in the control group who died before the index date were excluded. Participants who had a previous history of renal stones were excluded from both the gallstone and control groups. A total of 703 participants were excluded in the gallstone group. A total of 87 gallstone patients were additionally excluded due to the insufficient matching of participants. Finally, 20,711 gallstone patients and 82,844 control I participants were included in this study (Fig. 1a).

#### Study II

Renal stone patients were matched 1:4 with the control II participants who were not diagnosed with renal stones from 2002 through 2013. The control group was extracted from the total population (n = 1,101,568). The matching factors were identical to those of study I (age, group, sex, income group, region of residence, and past medical histories [hypertension, diabetes, and dyslipidemia]). The matching procedures and exclusion criteria were also identical to those of study I. A total of 473 participants were excluded in the renal stone group. An additional 35 renal stone patients were excluded due to insufficient matching of participants. Eventually, = 23,615 renal stone patients and 94,460 control II participants were analyzed in study II (Fig. 1b).

### Variables

Age was divided into 5-year intervals: 0–4, 5–9, 10–14, …, and 85+ years old. A total of 18 age groups were classified. The income groups were composed with 41 classes (one health aid class, 20 self-employment health insurance classes, and 20 employment health insurance classes). These groups were reconstituted to 11 classes (class 1 [lowest income]-11 [highest income]). The 16 regions of residence were classified according to administrative districts. These regions were recategorized into urban (Seoul, Busan, Daegu, Incheon, Gwangju, Daejeon, and Ulsan) and rural (Gyeonggi, Gangwon, Chungcheongbuk, Chungcheongnam, Jeollabuk, Jeollanam, Gyeongsangbuk, Gyeongsangnam, and Jeju).

The past medical histories were investigated based on ICD-10 codes. For the strict disease criteria, hypertension (I10 and I15), diabetes (E10-E14), and dyslipidemia (E78) were included if the participants had visited a hospital or clinical with that diagnosis ≥2 times.

### Statistical Analyses

The rates of general characteristics were compared between the gallstone and control groups (study I) and between the renal stone and control groups (study II) using the chi-square test.

In study I, the hazard ratio (HR) of gallstones (independent variable) for renal stones (dependent variable) was analyzed using a Cox proportional hazards model. In study II, the HR of renal stones (independent variable) for gallstones (dependent variable) was analyzed using another Cox proportional hazards model. For each analysis, crude (simple) and adjusted (age, sex, income, region of residence, hypertension, diabetes, and dyslipidemia) models were applied, and the 95% confidence intervals (CIs) were calculated.

For the subgroup analysis, the participants were divided according to age and sex (0–29 years old, 30–59 years old, 60+ years old; men, and women).

Two-tailed analyses were performed, and P values less than 0.05 were considered to designate significance. SPSS v. 21.0 (IBM, Armonk, NY, USA) was used for statistical analyses.

## Supplementary information

Supplementary Table S1

## References

[1] Gurusamy KS, Davidson BR (2014). Gallstones. BMJ.

[2] Everhart JE, Khare M, Hill M, Maurer KR (1999). Prevalence and ethnic differences in gallbladder disease in the United States. Gastroenterology.

[3] Jung HW, Chun KS, Kim YS, Kim MH, Choi H (1992). Prevalence of gallstones in Korean. Korean Acad Fam Med.

[4] Hahm JS (2003). Prevalence of gallstone disease in patients with end-stage renal disease treated with hemodialysis in Korea. Hepatogastroenterology.

[5] Kim MH (1999). Epidemiological study on Korean gallstone disease: a nationwide cooperative study. Dig Dis Sci.

[6] Pak M, Lindseth G (2016). Risk Factors for Cholelithiasis. Gastroenterol Nurs.

[7] Scales CD, Smith AC, Hanley JM, Saigal CS (2012). Urologic Diseases in America, P. Prevalence of kidney stones in the United States. Eur Urol.

[8] Kim H (2002). Prevalence and epidemiologic characteristics of urolithiasis in Seoul, Korea. Urology.

[9] Bae SR (2014). The epidemiology of reno-ureteral stone disease in Koreans: a nationwide population-based study. Urolithiasis.

[10] Pietrow PK, Karellas ME (2006). Medical management of common urinary calculi. Am Fam Physician.

[11] Romero V, Akpinar H, Assimos DG (2010). Kidney stones: a global picture of prevalence, incidence, and associated risk factors. Rev Urol.

[12] Acar B, Inci Arikan F, Emeksiz S, Dallar Y (2008). Risk factors for nephrolithiasis in children. World J Urol.

[13] Frassetto L, Kohlstadt I (2011). Treatment and prevention of kidney stones: an update. Am Fam Physician.

[14] Li CH, Sung FC, Wang YC, Lin D, Kao CH (2014). Gallstones increase the risk of developing renal stones: a nationwide population-based retrospective cohort study. QJM.

[15] Taylor EN, Chan AT, Giovannucci EL, Curhan GC (2011). Cholelithiasis and the risk of nephrolithiasis. J Urol.

[16] Akoudad S (2010). Correlates of kidney stone disease differ by race in a multi-ethnic middle-aged population: the ARIC study. Prev Med.

[17] Tsai CJ, Leitzmann MF, Willett WC, Giovannucci EL (2004). Prospective study of abdominal adiposity and gallstone disease in US men. Am J Clin Nutr.

[18] Jeong IG (2011). Association between metabolic syndrome and the presence of kidney stones in a screened population. Am J Kidney Dis.

[19] Nepokroeff CM, Lakshmanan MR, Ness GC, Dugan RE, Porter JW (1974). Regulation of the diurnal rhythm of rat liver beta-hydroxy-beta-methylglutaryl coenzmye A reductase activity by insulin, glucagon, cyclic AMP and hydrocortisone. Arch Biochem Biophys.

[20] Chait A, Bierman EL, Albers JJ (1979). Low-density lipoprotein receptor activity in cultured human skin fibroblasts. Mechanism of insulin-induced stimulation. J Clin Invest.

[21] Pak CY (2003). Biochemical profile of stone-forming patients with diabetes mellitus. Urology.

[22] Daudon M, Jungers P (2007). Diabetes and nephrolithiasis. Curr Diab Rep.

[23] Serrano PE, Khuder SA, Fath JJ (2010). Obesity as a risk factor for nosocomial infections in trauma patients. J Am Coll Surg.

[24] Rink RD, Kaelin CR, Giammara B, Fry DE (1981). Effects of live Escherichia coli and Bacteroides fragilis on metabolism and hepatic pO2. Circ Shock.

[25] Van Erpecum KJ (2011). Pathogenesis of cholesterol and pigment gallstones: an update. Clin Res Hepatol Gastroenterol.

[26] Haidinger, G. *et al*. Risk factors for lower urinary tract symptoms in elderly men. For the Prostate Study Group of the Austrian Society of Urology. *Eur Urol* **37**, 413–420, doi:20162 (2000). 10.1159/000020162 10765071

[27] Asplin JR (2002). Hyperoxaluric calcium nephrolithiasis. Endocrinol Metab Clin North Am.

[28] Meyer G (2005). Ion transport across the gallbladder epithelium. Curr Drug Targets Immune Endocr Metabol Disord.

[29] Choi HG, Park B, Sim S, Ahn SH (2016). Tonsillectomy Does Not Reduce Upper Respiratory Infections: A National Cohort Study. PLoS One.

[30] Kim SY (2017). Severe hearing impairment and risk of depression: A national cohort study. PLoS One.

[31] Kim MS, Kim SY, Kim JH, Park B, Choi HG (2017). Depression in breast cancer patients who have undergone mastectomy: A national cohort study. PLoS One.

[32] National Health Insurance Sharing Service, http://nhiss.nhis.or.kr/ (2015).

