# Supplementary material for: Bidirectional association between gallstones and renal stones: Two longitudinal follow-up studies using a national sample cohort
Source: Sci Rep. 2019 Feb 22;9:2620. doi: 10.1038/s41598-019-38964-2 (PMC6385337; doi:10.1038/s41598-019-38964-2)
Supplement: Supplementary file 1 — Supplementary Table S1 [file 41598_2019_38964_MOESM1_ESM.docx]

**Bidirectional association between gallstones and renal stones: Two longitudinal follow-up studies using a national sample cohort**

So Young Kim, MD^1¶^, Chang Myeon Song, MD^2¶^, Hyun Lim, MD^3^, Man Sup Lim, MD^4^, Woojin Bang, MD^5^, Hyo Geun Choi, MD^6*^

^1^Department of Otorhinolaryngology-Head & Neck Surgery, CHA Bundang Medical Center, CHA University, Seongnam, Korea

^2^Department of Otorhinolaryngology-Head & Neck Surgery, Hanyang University College of Medicine, Seoul, Korea

^3^Department of Internal Medicine, Hallym University College of Medicine, Anyang, Korea

^4^Department of General Surgery, Hallym University College of Medicine, Chuncheon, Korea

^5^Department of Urology, Hallym University College of Medicine, Anyang, Korea

^6^Department of Otorhinolaryngology-Head & Neck Surgery, Hallym University College of Medicine, Anyang, Korea

^¶^These authors equally contributed to this study

*Correspondence: [pupen@naver.com](mailto:pupen@naver.com)

**Supplementary Table S1** Analysis of crude and adjusted hazard ratios (95% confidence interval) of renal stone in gallstone (study I) and of gallstone in renal stone (study II) in the period > 6 months after the index date.

| Characteristics | | Hazard ratios | | | |
| --- | --- | --- | --- | --- | --- |
|  |  | Crude | P-value | Adjusted† | P-value |
| Study I | | | | | |
|  | Gallstone | 1.44 (1.27-1.62) | <0.001* | 1.43 (1.27-1.61) | <0.001* |
|  | Control | 1.00 |  | 1.00 |  |
| Study II | | | | | |
|  | Renal stone | 1.54 (1.40-1.70) | <0.001* | 1.53 (1.39-1.69) | <0.001* |
|  | Control | 1.00 |  | 1.00 |  |

* Cox proportional hazard regression model, significance at P < 0.05

† Adjusted model for age, sex, income, region of residence, hypertension, diabetes, and dyslipidemia
